# Publisher Correction: Prominence of the tropics in the recent rise of global nitrogen pollution

**DOI:** 10.1038/s41467-019-13567-7

**Published:** 2019-12-04

**Authors:** Minjin Lee, Elena Shevliakova, Charles A. Stock, Sergey Malyshev, P. C. D. Milly

**Affiliations:** 1 Program in Atmospheric and Oceanic Sciences, Princeton University, 300 Forrestal Road, Sayre Hall, Princeton, NJ 08540 USA; 2 NOAA/Geophysical Fluid Dynamics Laboratory, 201 Forrestal Road, Princeton, NJ 08540 USA; 3 U.S. Geological Survey and NOAA/Geophysical Fluid Dynamics Laboratory, 201 Forrestal Road, Princeton, NJ 08540 USA

**Keywords:** Element cycles, Element cycles, Element cycles

Correction to: *Nature Communications* 10.1038/s41467-019-09468-4, published online 29 March 2019.

The original version of this Article contained errors in the References.

The citation currently given as reference [13] was previously incorrectly given as reference [14], and vice versa.

The citation currently given as reference [31] was previously incorrectly given as reference [37]. As a consequence of this, current references [32–37] were previously incorrectly numbered [31–36], respectively.

This has been corrected in both the PDF and HTML versions of the Article.

