# Supplementary material for: Prominence of the tropics in the recent rise of global nitrogen pollution
Source: Nat Commun. 2019 Mar 29;10:1437. doi: 10.1038/s41467-019-09468-4 (PMC6440957; doi:10.1038/s41467-019-09468-4)
Supplement: Supplementary file 1 — Supporting Information [file 41467_2019_9468_MOESM1_ESM.pdf]

Nature Communications

Supplementary Information for

Prominence of the tropics in the recent rise of global nitrogen pollution

Minjin Lee et al.

Correspondence to: minjinl@princeton.edu

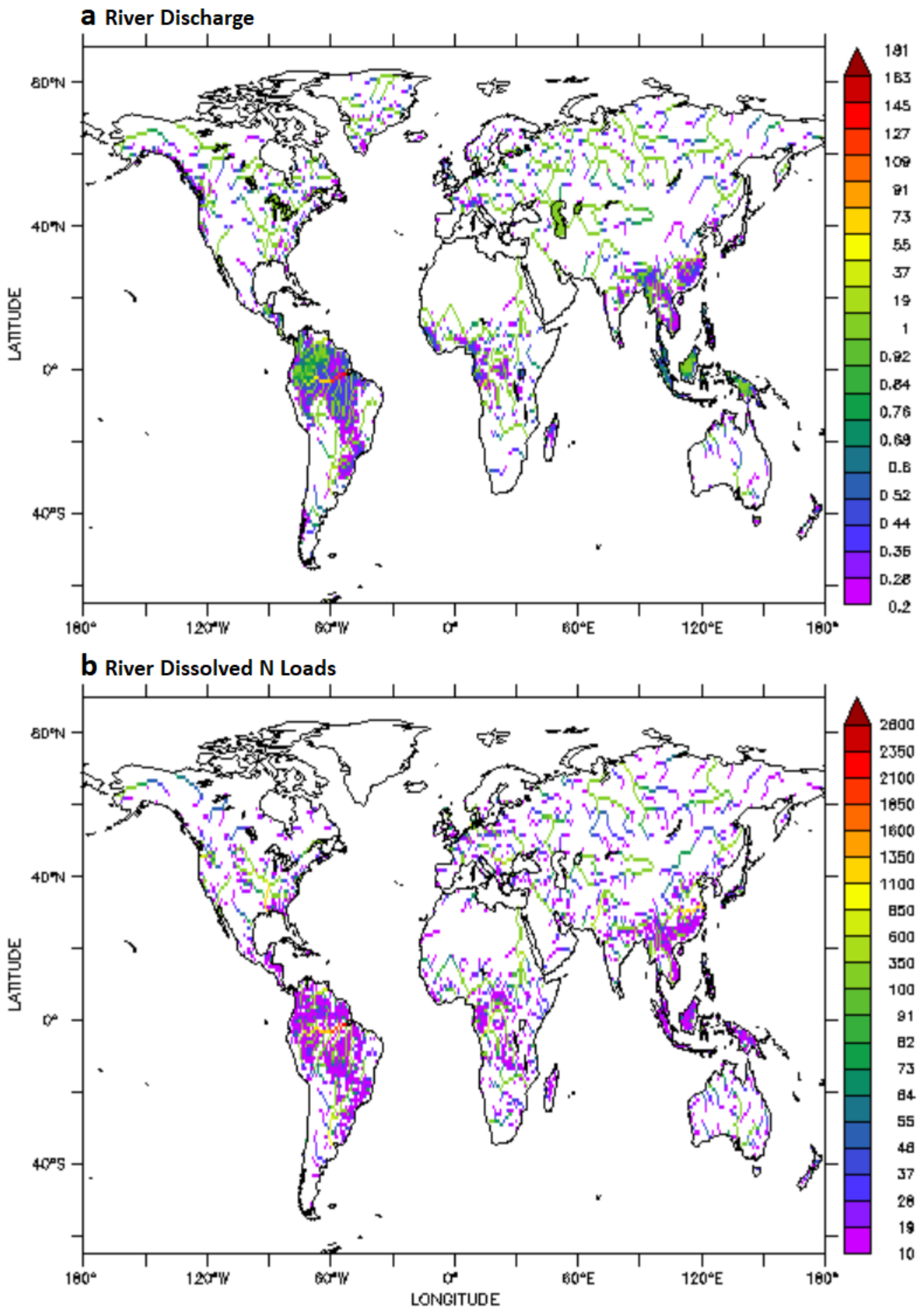

Supplementary Figure 1. River discharge and dissolved N loads. a, b, Simulated river discharge (10^3 m^3 s^{-1}) (**a**) and dissolved N (DN) loads (ktN yr^{-1}) (**b**) for the year 1995.

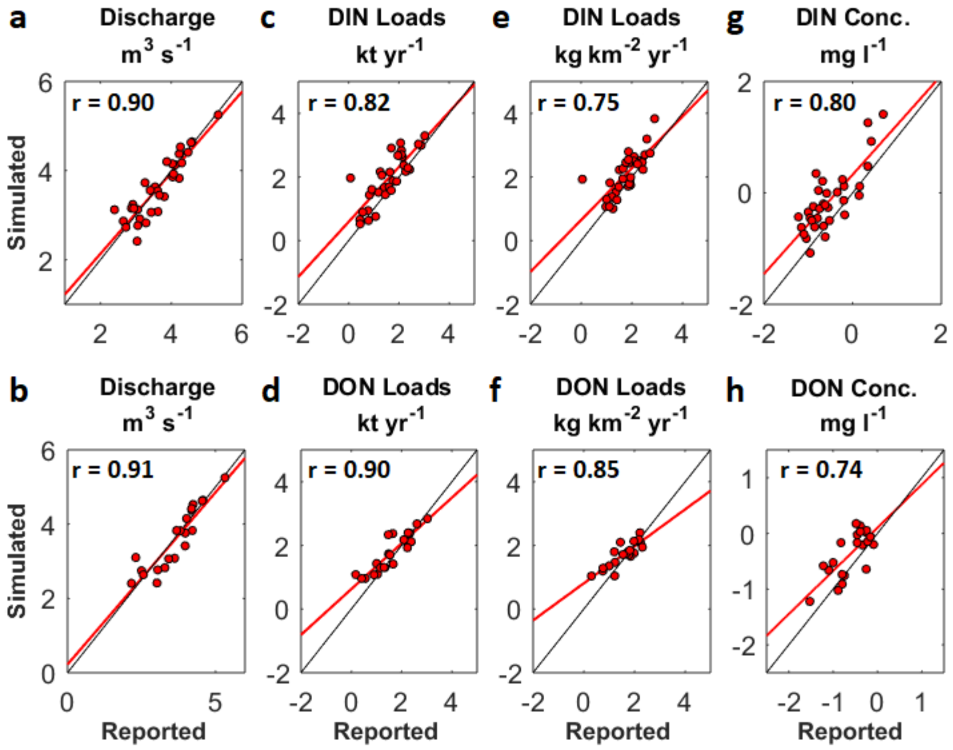

Supplementary Figure 2. Reported and simulated river discharge and N loads. LM3-TAN results were compared with reported river discharge and measurement-based estimates of dissolved organic N (DON) loads and concentrations from 21 large rivers[1-2] and reported river discharge and measurement-based estimates of dissolved inorganic N (DIN) loads and concentrations from 33 large rivers[2-3] for the year 1995 (Supplementary Table 2). **a, b,** discharge in m^3 s^{-1} (Log-scale); **c, d,** DIN and DON loads in kt yr^{-1} (Log-scale); **e, f,** DIN and DON loads in kg km^{-2} yr^{-1} (Log-scale); **g, h,** DIN and DON concentrations in mg l^{-1} (Log-scale).

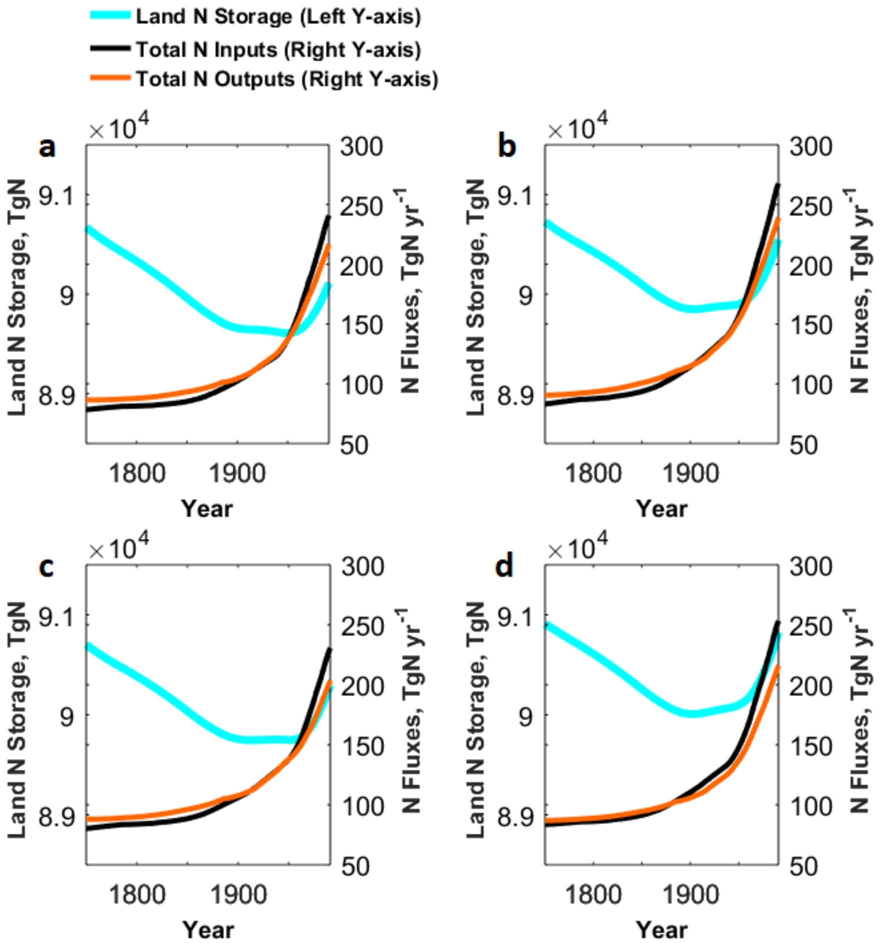

Supplementary Figure 3. Global land N storage and fluxes under different scenarios. a-d, Land N storage (cyan), total Nr inputs (black), and total N outputs (orange) in simulations with BNF settings spanning the upper (**a**) and lower (**b**) bounds of the published ranges[4-5], different fertilizer inputs[6] (**c**), and a different LULUC scenario without shifting cultivation[7] (**d**) (See Methods). All plots show 30-year moving averages from 1750 to 2005.

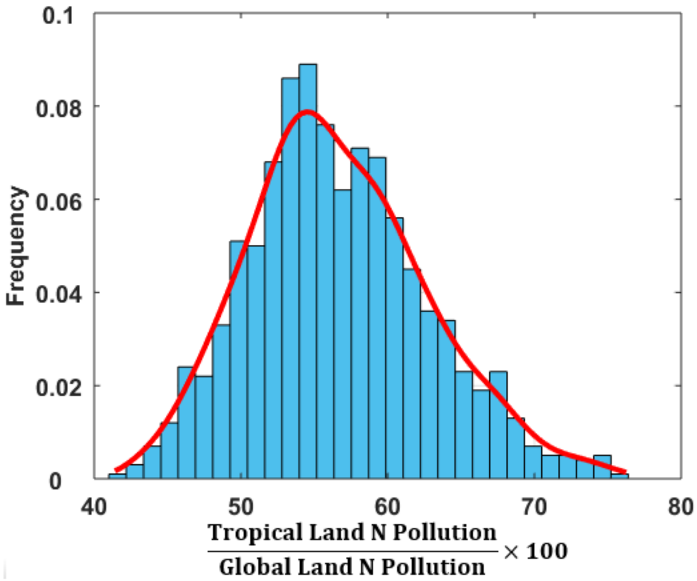

Supplementary Figure 4. Prominence of the tropics in global N pollution. Tropical (global) land N pollution was estimated as the sum of N outputs from tropical (global) land, minus the sum of environmentally benign portions of the outputs (See Methods and Supplementary Note 2). These estimates from the no CO_2 fertilization simulation suggest that the tropics create 57±6% of global land N pollution despite covering only 34% of global land area and receiving far lower amounts of synthetic fertilizers than the extratropics. The reported uncertainties consider the sensitivity of the pollution estimates to different partitioning of the outputs into environmentally benign vs. pollutant forms.

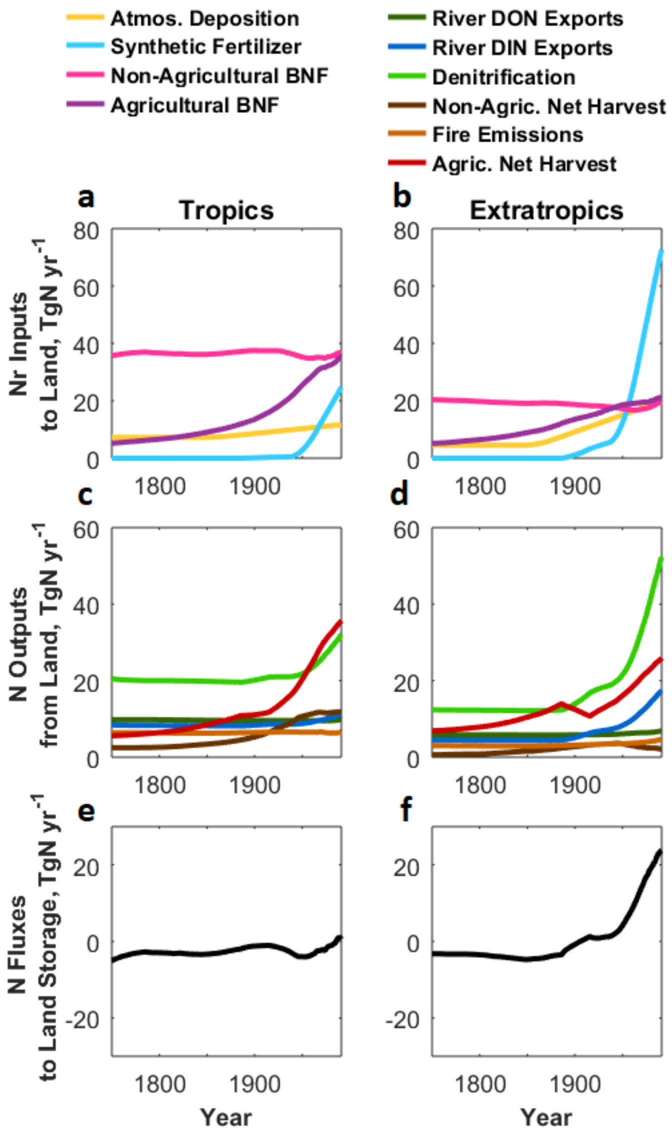

Supplementary Figure 5. Land N fluxes in the tropics and extratropics. This figure shows results, which were simulated by using the low BNF setting (See Methods and Supplementary Table 3). **a, b,** Land Nr inputs include atmospheric deposition (light orange), synthetic fertilizers (sky-blue), biological N fixation (BNF) in non-agricultural (plum) and agricultural (purple) lands. **c, d,** Land N outputs include river dissolved organic N (DON) exports (green), river dissolved inorganic N (DIN) exports (blue), soil and freshwater denitrification (light green), fire emissions (orange), net harvest in agricultural (red) and non-agricultural (brown) lands. **e, f,** N fluxes to land storage. All plots show 30-year moving averages from 1750 to 2005.

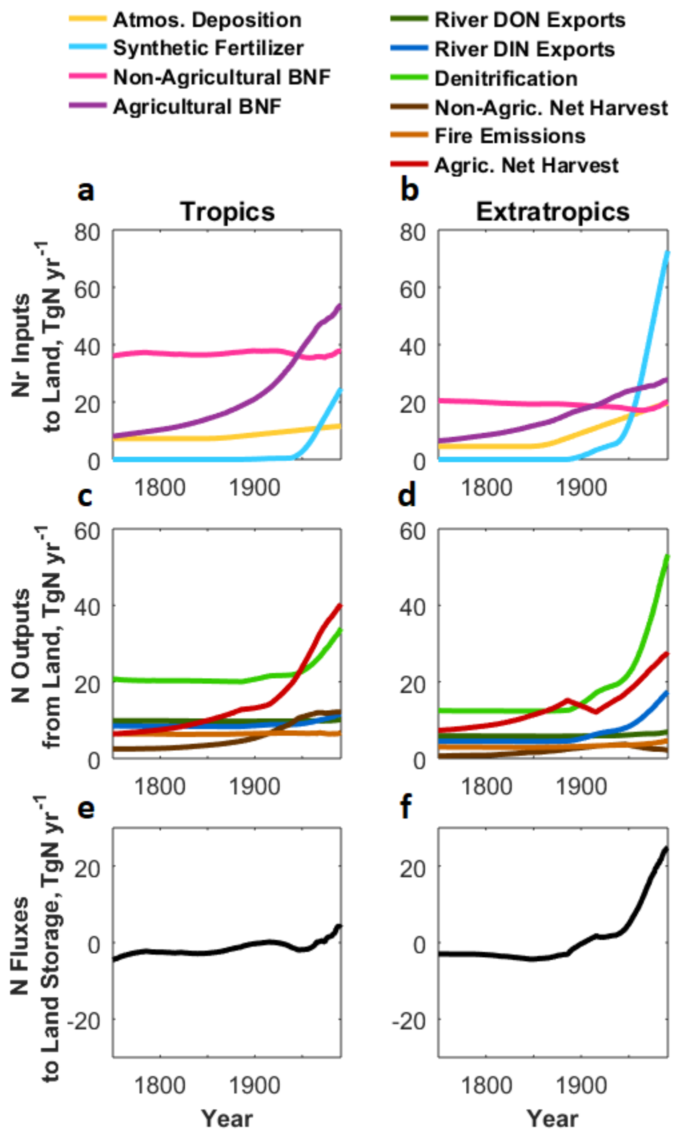

Supplementary Figure 6. Land N fluxes in the tropics and extratropics. This figure shows results, which were simulated by using the high BNF setting (See Methods and Supplementary Table 3). **a, b,** Land Nr inputs include atmospheric deposition (light orange), synthetic fertilizers (sky-blue), biological N fixation (BNF) in non-agricultural (plum) and agricultural (purple) lands. **c, d,** Land N outputs include river dissolved organic N (DON) exports (green), river dissolved inorganic N (DIN) exports (blue), soil and freshwater denitrification (light green), fire emissions (orange), net harvest in agricultural (red) and non-agricultural (brown) lands. **e, f,** N fluxes to land storage. All plots show 30-year moving averages from 1750 to 2005.

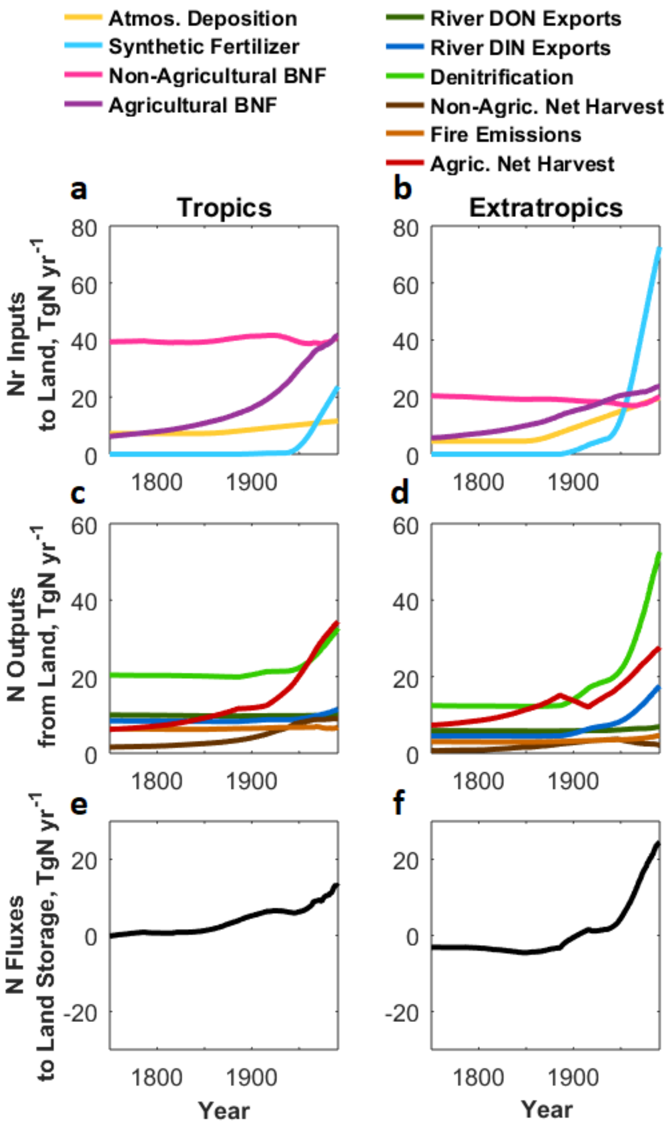

Supplementary Figure 7. Land N fluxes in the tropics and extratropics. This figure shows results, which were simulated by using a LULUC scenario without shifting cultivation[7]. **a, b,** Land Nr inputs include atmospheric deposition (light orange), synthetic fertilizers (sky-blue), biological N fixation (BNF) in non-agricultural (plum) and agricultural (purple) lands. **c, d,** Land N outputs include river dissolved organic N (DON) exports (green), river dissolved inorganic N (DIN) exports (blue), soil and freshwater denitrification (light green), fire emissions (orange), net harvest in agricultural (red) and non-agricultural (brown) lands. **e, f,** N fluxes to land storage. All plots show 30-year moving averages from 1750 to 2005.

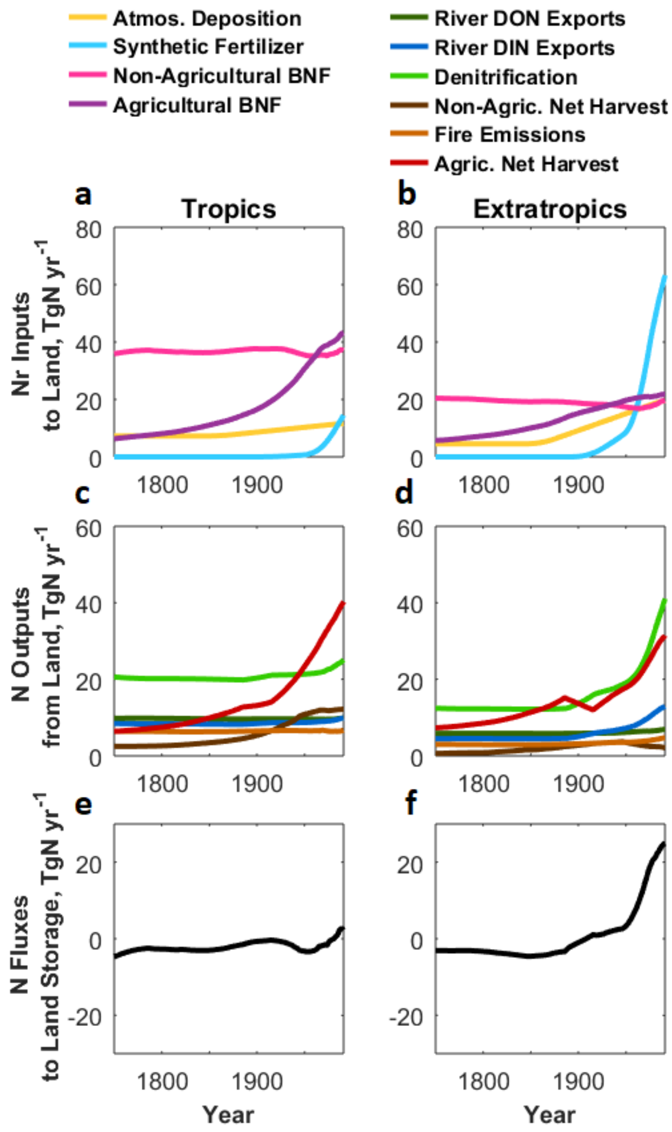

Supplementary Figure 8. Land N fluxes in the tropics and extratropics. This figure shows results, which were simulated by using fertilizer inputs from Lu and Tian[6]. **a, b,** Land Nr inputs include atmospheric deposition (light orange), synthetic fertilizers (sky-blue), biological N fixation (BNF) in non-agricultural (plum) and agricultural (purple) lands. **c, d,** Land N outputs include river dissolved organic N (DON) exports (green), river dissolved inorganic N (DIN) exports (blue), soil and freshwater denitrification (light green), fire emissions (orange), net harvest in agricultural (red) and non-agricultural (brown) lands. **e, f,** N fluxes to land storage. All plots show 30-year moving averages from 1750 to 2005.

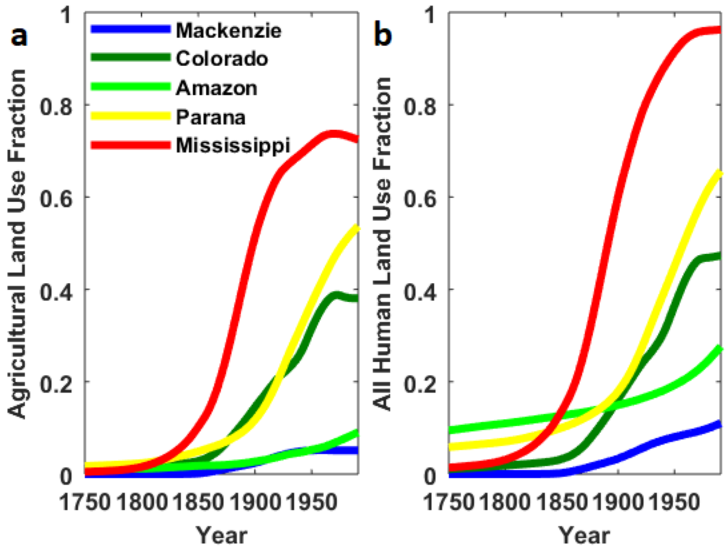

Supplementary Figure 9. Historical human land-use changes. Thirty-year moving averages of historical (1750-2005) land-use fraction changes for the Mackenzie (blue), Colorado (blue-green), Amazon (green), Parana (yellow), and Mississippi (red) River Basins. **a,** Agricultural land use (i.e., cropland and pasture); **b,** All land use disturbed by human activities (i.e., agricultural land use and secondary land use – abandoned agricultural land or regrowing forest after logging).

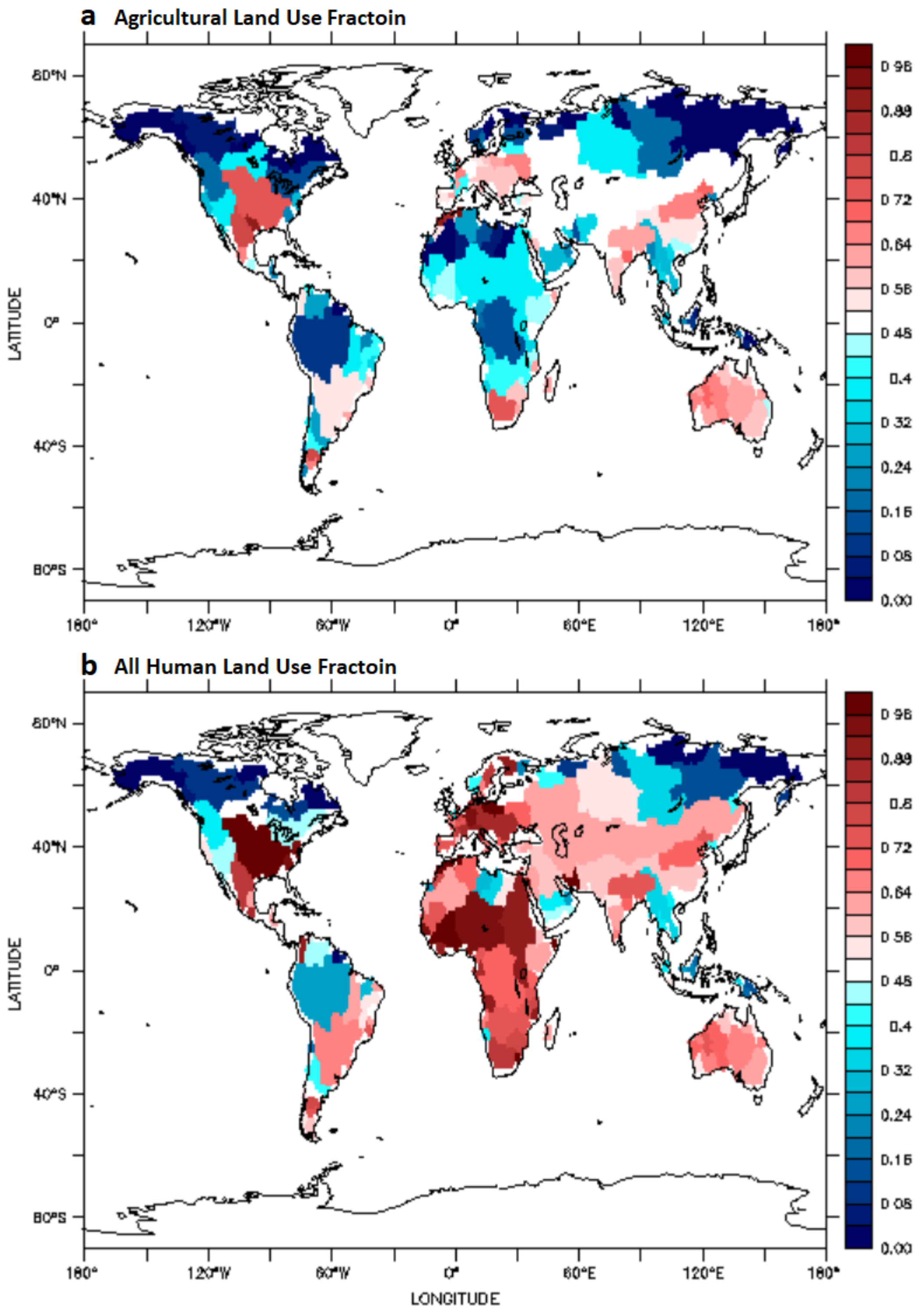

Supplementary Figure 10. Global distributions of historical human land use. Contemporary (1976-2005 mean) basin-wide mean fraction of human land use for 159 globally-distributed major river basins. **a,** Agricultural land use (cropland and pasture); **b,** All land use disturbed by human activities (i.e., agricultural land use and secondary land use (abandoned agricultural land or regrowing forest after logging)).

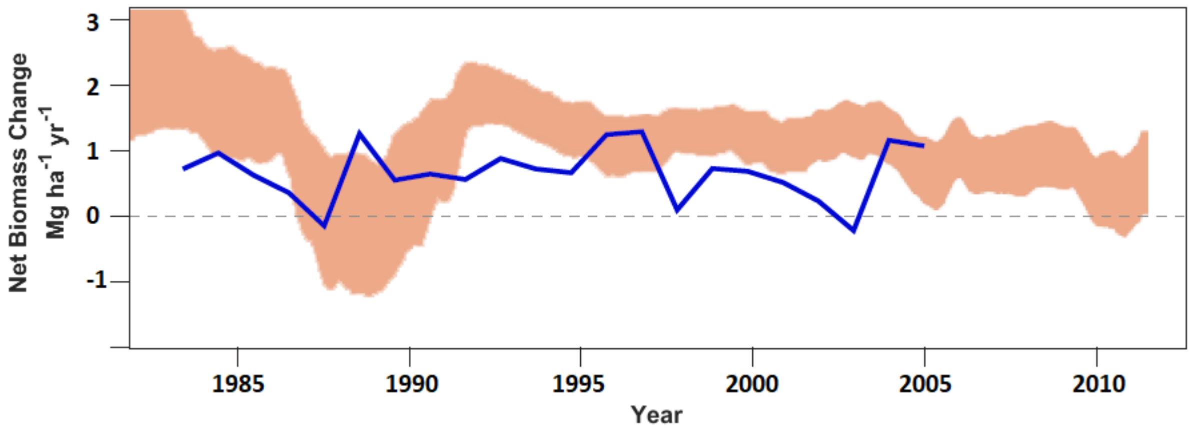

Supplementary Figure 11. A long-term increase in the aboveground biomass density in Amazonian intact forests. Our simulation shows positive net biomass changes in Amazonian intact forests during 1983-2005 (blue solid line), which are consistent with corresponding uncertainty bounds (shaded orange area) reported in Brienen and colleagues[8].

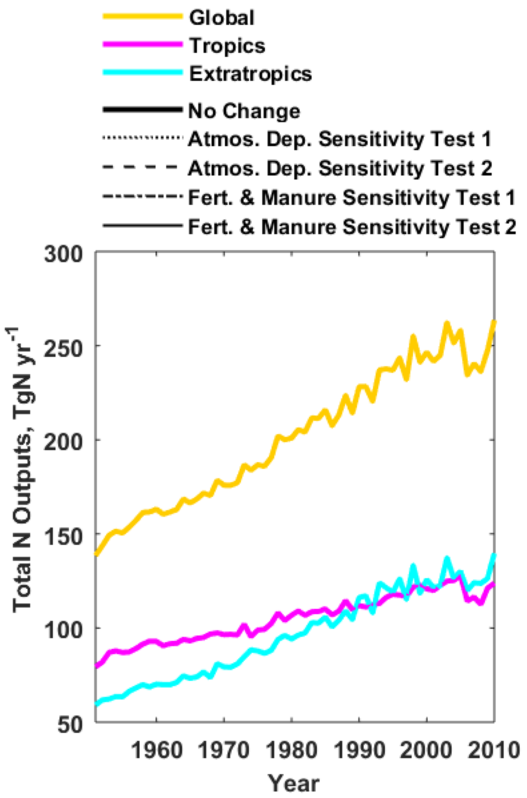

Supplementary Figure 12. Sensitivity tests of different fractions to divide Nr inputs into three N species. Each of Nr inputs was divided into three N species (i.e., organic, ammonium, and nitrate plus nitrite N) by multiplying reported fractions (See Methods and Supplementary Table 4)[9-10]. Sensitivity tests suggest that changing fractionation of N species has almost no influence on total land N outputs from global, tropical, and extratropical lands.

		LM3-TAN 1991-2005 average	Published Estimates 1990s, [2000s]
a	Biological N fixation (BNF)	128 (116-145)	[1]112, [2]139
	Agricultural	69 (59-85)	[2]32, [[3]50-70]
	Preindustrial	76 (73-81)	[4]58 (40-100), [5]195 (100-290)
	Non-agricultural[A]<br>Natural	59 (58-62)	 [2]107, [6]128
b	Atmospheric deposition	[1]32	[2]59
c	Haber-Bosch (Synthetic fertilizers)	[7]114 ([7,8]88[B])	[2]100, [[9]120]
d	Fluxes to the ocean	49 (42-50)	[2]48, [[10,11]45]
e	Fluxes to the atmosphere	178 (150-198)	[2]189
f	Denitrification N_2 emissions	104 (73-116)[C]	[2]115[D], [[12]96[D]]
g	Other emissions	74 (77-82) [E]	[2]74[F], [13,14] [70 (60-82)[G]]
h	Fluxes to the land storage	32 (28-56)	[2]60, [[15]27]
i	Soils/litter storage	86124 (85924-86505)	[16]95000 (70000-820000)

[A]Primary plus secondary lands. (See Methods for a land-use description.)
[B]The sum of Lu and Tian[6]'s fertilizer to croplands and Bouwman and colleagues[15]'s fertilizer allocated to grasslands in mixed systems. (See Methods for model forcing and simulations.)
[C]Upper bound on total denitrification N_2 emissions. These fluxes are likely reduced by denitrification nitrous oxide (N_2O) emissions. (See Supplementary Note 2.)
[D]Total denitrification N_2 emissions.
[E]Lower bound on other emissions of NO_x, NH_3, and N_2O. These fluxes are likely enhanced by denitrification N_2O emissions. (See Supplementary Note 2.)
[F]NO_x and NH_3 emissions from food, energy, and natural sources, except combustion processes that create new Nr (See Table 2 in Galloway and colleagues[4]) plus N_2O emissions from soils and rivers (See Table 3 in Galloway and colleagues[4]).
[G]NO_x and NH_3 emissions from agriculture, biomass and biofuel burning, and soils under natural vegetation plus N_2O emissions from agriculture, biomass and biofuel burning, human excreta, and soils under natural vegetation (See Table 6.9 in Ciais and colleagues[20]).

Supplementary Table 1. Comparison of simulated global land N budgets with published estimates. References are marked as superscript: 1, Green and colleagues[5]; 2, Galloway and colleagues[4]; 3, Herridge and colleagues[11]; 4, Vitousek and colleagues[12]; 5, Cleveland and colleagues[13]; 6, Cleveland and colleagues[14]; 7, Bouwman and colleagues[15]; 8, Lu and Tian[6]; 9, Galloway and colleagues[16]; 10, Mayorga and colleagues[17]; 11, Seitzinger and colleagues[18]; 12, Bouwman and colleagues[19]; 13, Ciais and colleagues[20]; 14, Dentener and colleagues[21]; 15, Zaehle[22]; 16, Post and colleagues[23] and references in Post and colleagues. Units are TgN yr^{-1} for fluxes and TgN for storage. The values in parentheses are the results with a range of BNF settings[4-5], different fertilizers[6,15], different LULUC[7], and different fractionation of N species in Nr inputs[9-10] (See Methods).

Continent	River	Basin Area km^2	River N Load kt yr^{-1}	River Discharge m^3 s^{-1}
**Reported river discharge and DON loads from 21 large rivers**				
South America	Amazon	6112000	1056	209315
Africa	Zaire	3698000	213	37524
North America	Mississippi	2926507	408	15776
South America	Parana	2783000	45	17650
Asia	Lena	2490000	241	16581
North America	Mackenzie	1787000	30	9633
Africa	Niger	1200000	20	4887
South America	Orinoco	1100000	181	35927
Asia	Ganges	1050000	30	15649
North America	St. Lawrence	1020000	10	10674
Africa	Orange	1000000	2	317
North America	Yukon	831387	171	9491
Europe	Danube	817000	123	6477
Asia	Kolyma	660000	46	4186
North America	Colorado	638951	4	203
North America	Rio Grande (TX)	456701	3	145
Asia	Khatanga	364000	34	2655
Asia	Indigirka	362000	22	1951
Asia	Yana	238000	11	1057
Asia	Olenek	219000	14	1111
North America	Brazos+Colorado (TX)	225355	8	365
**Reported river discharge and DIN loads from 33 large rivers**				
South America	Amazon	6112000	1054	208968
Africa	Zaire	3698000	120	38052
Asia	Ob	2990000	267	12811
North America	Mississippi	2980000	762	16774
South America	Parana	2783000	125	18011
Asia	Yenisei	2590000	186	19660
Asia	Lena	2490000	58	16648
Asia	Amur	1855000	155	10908
Asia	Chang Jiang	1808000	594	29427
Africa	Zambezi	1330000	18	3361
South America	Orinoco	1100000	136	35991
Australia	Murray	1060000	1	251
North America	St. Lawrence	1020000	81	10686
Asia	Indus	916000	125	1807
North America	Yukon	849000	26	6342
Asia	Huang He	752000	91	1300
North America	Columbia	669000	50	7484
Asia	Kolyma	660000	12	4186

Asia	Zhujiang	437000	229	11511
Asia	Khatanga	364000	6	2705
Asia	Indigirka	362000	4	1934
Europe	N. Dvina	348000	15	3488
Europe	Pechora	324000	29	4154
North America	Churchill (Hudson Bay)	298000	3	819
Europe	Neva	282000	21	2549
South America	Uruguay	240000	41	4598
Asia	Yana	238000	6	1088
Asia	Olenek	219000	3	1135
Africa	Rufiji	178000	49	1116
Europe	Elbe	146000	116	752
Asia	Chao Phraya	114000	7	882
North America	Balsas	112000	8	444
Europe	Odra	112000	44	526

Supplementary Table 2. Reported river discharge and N loads. LM3-TAN results were compared with reported river discharge and measurement-based estimates of dissolved organic N (DON) loads and concentrations from 21 large rivers[1-2] and reported river discharge and measurement-based estimates of dissolved inorganic N (DIN) loads and concentrations from 33 large rivers[2-3] for the year 1995 (Supplementary Figures 1 and 2). We chose the rivers of which basin areas are larger than 100,000 km^2 and $\log_{10}$ of their discharges are larger than 2.2.

Parameter	Description	Value	Unit	Reference or Rationale
**Parameters in the terrestrial component equations**				
$r_{DOM}, r_{NH_4^+}, r_{NO_3^-}$	calibration factors for dissolved organic matter, ammonium N, and nitrate N	1, 1, 50	unitless	calibrated to match stream N loads adjusted from Lee and colleagues[24]
f_{DOM}	fraction of litter soil decomposition that becomes potential DOM	0.2	unitless	calibrated to match stream DON loads adjusted from Gerber and colleagues[25]
k_{SS}	decomposition rate of slow soil	0.03	1/year	Parton and colleagues[26] adjusted from Gerber and colleagues[25]
r_{SS}	C:N ratio of slow soil	13.3	unitless	Post and colleagues[23] adjusted from Gerber and colleagues[25]
$r_{L,tropical}$	C:N ratio in tropical vegetation leaves	25	unitless	Cernusak and colleagues[27] adjusted from Gerber and colleagues[25]
l_{BNF}	upper limit of biological fixation	60 (40-120)	kg/ha/yr	Cleveland and colleagues[13] newly introduced
**Parameters in the freshwater component equations**				
$k'_{denitr,min}$	minimum reaction rate constant of river denitrification	0.034/86400	$1/s$	Alexander and colleagues[28] adjusted from Lee and colleagues[24]
$k''_{denit,min}$	minimum reaction rate constant of lake denitrification	0.034/86400	$1/s$	Alexander and colleagues[28] newly introduced
$k''_{denit,max}$	maximum reaction rate constant of river denitrification	0.05/86400	$1/s$	calibrated to match river N exports newly introduced
k''_{min}, k''_{nit}	reaction rate constants of lake mineralization and nitrification	0.11/86400<br>0.51/86400	$1/s$	calibrated to match lake mineralization and nitrification newly introduced
T''_p	parameter	1.047	unitless	Wade and colleagues[29] newly introduced
T''_r	Reference lake water temperature	20	℃	Wade and colleagues[29] newly introduced

Supplementary Table 3. Newly introduced or adjusted parameters from the earlier developments. The calibration factors were introduced by Lee and colleagues[24] to compensate for processes that were not accounted for in the model, such as impacts of soil microbes and N storage in lentic systems. The incorporation of the lake N cycle into the model led these parameters to be adjusted at lower values than in the previous development. Uncertainty tests of low and high BNF (116 and 145 TgN yr^{-1}) were simulated by setting an upper limit of BNF as 40 and 120 kgN ha^{-1} yr^{-1} respectively (See Supplementary Table 1 and Supplementary Note 1).

	Organic N	Ammonium N	Nitrate plus nitrite N
Atmos. dep.	0	49.0	51.0
Atmos. dep. sensitivity test 1	0	62.0	38.0
Atmos. dep. sensitivity test 2	0	36.0	64.0
Fertilizers and manure	35.8	51.1	13.1
Fertilizers and manure sensitivity test 1	26.0	64.0	10.0
Fertilizers and manure sensitivity test 2	45.0	38.0	17.0
Urban wastewaters	63.4	21.3	15.3

Supplementary Table 4. Reported fractions to divide N inputs into three N species. Atmospheric deposition was divided into ammonium and nitrate plus nitrite N by using reported fractions of multi-model mean N deposition from the Atmospheric Chemistry and Climate Model Intercomparison Project[9]. For fertilizers, manure, and urban wastewater, reported input fractions for the Chesapeake Bay Program's Watershed Model were adopted[10]. We did not conduct sensitivity tests for urban wastewaters, because their amount is very small compared to the other N sources. Sensitivity tests show that different fractions (i.e., -25% and +25% fractions of the dominant N species for each N source) have almost no influence on land N storage and fluxes (Supplementary Figure 12).

Supplementary Note 1. Comparison of simulated global land nitrogen (N) budgets with published estimates. We compare simulated global land N storage and fluxes with published estimates in 16 different studies[4-6,11-23] (Supplementary Table 1). We focus on agreement during the contemporary period, which is of primary interest to the results of this paper and for which more data is available. While published N storage and flux estimates are invaluable in identifying and understanding the dominant global N cycling processes, the authors of these studies also acknowledge considerable uncertainties due to sparse measurements and subsequent assumptions required to estimate the global magnitude of different N cycling processes. We thus emphasize consistency in the magnitude and direction of N storage and fluxes.

Simulated contemporary BNF in our baseline simulation (128 TgN yr^{-1}) is between 112 TgN yr^{-1} estimated by Green and colleagues[5] and 139 TgN yr^{-1} estimated by Galloway and colleagues[4] (Supplementary Table 1a). Simulated preindustrial BNF (76 TgN yr^{-1}) is well within the latest estimate of 58 (40-100) TgN yr^{-1} by Vitousek and colleagues[12]. Simulated BNF in agricultural lands (cropland and pasture; 69 TgN yr^{-1}) is within the latest BNF estimate in agricultural systems (50-70 TgN yr^{-1}) by Herridge and colleagues[11]. Our estimate lies on the high end of Herridge and colleagues[11]'s range, in part, because the agricultural land area simulated by using the LULUC scenarios of Hurtt and colleagues[7] (49 $10^6 km^2$) is larger than the area considered to calculate the Herridge and colleagues[11]'s estimate (27 $10^6 km^2$). See Methods for a land-use description.

Simulated BNF in non-agricultural (primary and secondary) lands (59 TgN yr^{-1}) is smaller than published BNF in natural systems by Galloway and colleagues[4] (107 TgN yr^{-1}) and by Cleveland and colleagues[14] (128 TgN yr^{-1}). In contrast to agricultural BNF, this is partly because the non-agricultural land area derived from Hurtt and colleagues[7]'s LULUC scenarios (82 $10^6 km^2$) is smaller than the area considered to calculate the Cleveland and colleagues[14]'s estimate (104 $10^6 km^2$). Furthermore, we note the suggestion by Vitousek and colleagues[12] that contemporary natural BNF is likely lower than their preindustrial estimate 58 (40-100) TgN yr^{-1} due to land conversion for cultivation and perhaps to downregulation of BNF under increasing anthropogenic Nr inputs. This implies that the estimates of Galloway and colleagues[4] and Cleveland and colleagues[14] may be high.

The range of published BNF estimates highlights the substantial uncertainty in this important element of the N budgets. As noted in the main text, we thus conducted uncertainty tests of low and high contemporary BNF (116 and 145 TgN yr^{-1}, see Supplementary Table 3). The results suggest that the different BNF settings have little effect on the patterns of global, extratropical, and tropical land N fluxes and pollution (Figures 3a and 4, Table 1, Supplementary Figures 3, 5, and 6).

Moving on to atmospheric N deposition, Galloway and colleagues[4] estimated 59 TgN yr^{-1} as the sum of atmospheric NO_y, (25 TgN yr^{-1}) and NH_x (34 TgN yr^{-1}) deposition to land, while Green and colleagues[5] estimated 32 TgN yr^{-1} (Supplementary Table 1b). We used the Green and colleagues[4]'s estimate of atmospheric deposition.

For fertilizer, we applied estimates from Bouwman and colleagues[15] (114 TgN yr^{-1}) that are between the Haber-Bosch estimate by Galloway and colleagues[4] (100 TgN yr^{-1} for 1990s) and that by Galloway and colleagues[16] (120 TgN yr^{-1} for 2000s) (Supplementary Table 1c). We also conducted an uncertainty test of different fertilizer by Lu and Tian[6]. Applications of the both fertilizers show higher land N sequestration in the extratropics than in the tropics (Figure 3a) and create similar global, extratropical, and tropical land N pollution and fluxes (Figures 3a and 4, Table 1, Supplementary Figures 3 and 8).

Simulated N fluxes to the ocean (49 TgN yr^{-1}) is consistent with 48 TgN yr^{-1} estimated by Galloway and colleagues[4] and 45 TgN yr^{-1} estimated by Mayorga and colleagues[17] and Seitzinger and colleagues[18] (Supplementary Table 1d). We further evaluate N fluxes to the ocean by comparing simulated regional

river dissolved inorganic and organic N loads and concentrations with measurement-based estimates from 47 major rivers, which are distributed broadly over the globe and influenced by various climates, biomes, and human activities[1-3] (Supplementary Figures 1 and 2, Supplementary Table 2). The correlations between the simulated and reported estimates of river discharge, N loads and concentrations all fall between 0.74 and 0.91.

Atmospheric N emissions in LM3-TAN arise from simulated denitrification (on land and within rivers and lakes), fire emissions, and harvested material, which is presumed to be primarily a precursor to atmospheric N pollution via various pathways including wood, biofuel, and waste burning, livestock respiration, emissions from food, human, and livestock waste[4,15,20]. As described in the main text and Methods, the portion of the harvest associated with atmospheric emissions is calculated as the harvest remaining after subtracting manure applications, wastewater discharges to rivers, and the fraction of the harvest used for durable goods (e.g., home building). Manure applications and wastewater discharges are specified model forcings (See Methods, Bouwman and colleagues[15], Van Drecht and colleagues[30]) and subtracted accordingly, while Zaehle[22] estimated human appropriation of N as 15 TgN yr^{-1}. This accounts for a 0.17 fraction of our net harvest, and we consider a range of 0.1-0.3 (Supplementary Note 2) to estimate LM3-TAN's total atmospheric N emissions of 178 (150-198) TgN yr^{-1}, which are consistent with 189 TgN yr^{-1} estimated by Galloway and colleagues[4] (Supplementary Table 1e).

LM3-TAN simulated soil and freshwater denitrification (N_2+N_2O) emissions of 96 (73-98) TgN yr^{-1}. This value would be further augmented by denitrification associated with harvested material. If we consider that 0.09 (0-0.2) of the harvested material is denitrified (See Supplementary Note 2), LM3-TAN produced a total denitrification estimate of 104 (73-116) TgN yr^{-1}. This compares favorably with denitrification N_2 emission estimates by Galloway and colleagues[4] (115 TgN yr^{-1}) and by Bouwman and colleagues[19] (96 TgN yr^{-1}) (Supplementary Table 1f). We note that the partitioning of total denitrification into N_2O (which is discussed in Supplementary Note 2 in the context of pollution estimates) is generally small relative to N_2 emissions[4,19-20,22] and does not affect the agreement in the magnitude of these estimates. Furthermore, subtracting total denitrification from total atmospheric emissions yields an estimate of atmospheric N emissions that are not associated with denitrification (74 (77-82) TgN yr^{-1}) which provides a lower bound on other emissions. These fluxes are likely enhanced by denitrification N_2O emissions (See Supplementary Note 2) and is consistent with emissions of 74 TgN yr^{-1} estimated by Galloway and colleagues[4] and 70 (60-82) TgN yr^{-1} estimated by Ciais and colleagues[20] and Dentener and colleagues[21] (Supplementary Table 1g).

Simulated N fluxes to the land storage is 32 TgN yr^{-1} (Supplementary Table 1h), which is similar in magnitude though less than 60 TgN yr^{-1} estimated by Galloway and colleagues[4] and consistent with estimates by other terrestrial ecosystem models (e.g., 27 TgN yr^{-1}; Zaehle[22]). Galloway and colleagues[4] and Gruber and Galloway[31] acknowledged significant uncertainty in their global estimate and the gap between our estimate and theirs could be easily closed by higher total land Nr inputs of Galloway and colleagues[4] (+22 Tg N yr^{-1}). Simulated global soils/litter N storage (86124 TgN) is within reported estimates (70000-820000 TgN) by Post and colleagues[23] and references in Post and colleagues[23] (Supplementary Table 1i).

Supplementary Note 2. Estimation of total land N pollution. Directly simulated N compartments and fluxes in LM3-TAN provide closed land N budgets and estimates of the total N fluxes from land to the atmosphere and ocean. Not all N fluxes, however, are harmful. N_2 is generally considered benign, as is N sequestered into durable goods (e.g., home building). It has furthermore been suggested that organic N exports from rivers to the coastal ocean are less harmful pollutants than inorganic N exports due to their relative long-lived nature[32]. This is, however, likely to only apply to acute local impacts at river mouths and does not preclude broader impacts on continental shelf and ocean scales. Total land N pollution for our study was thus estimated as total land N outputs, minus the sum of N_2 emissions and human appropriation of the net harvest into durable goods.

To estimate total land N pollution, we did three additional partitioning of the fluxes directly simulated by LM3-TAN: 1) the partitioning of the soil and freshwater denitrification into N_2O and N_2 emissions, 2) the partitioning of the net harvest into N_2 emissions, and 3) the partitioning of the net harvest transformed into durable goods. To test the robustness of our results to uncertainty in these partitions, an interval for each partition was assigned based on the scientific literature and 1000 Monte Carlo style calculations were conducted with random draws from a uniform distribution across the uncertainty interval. This was done for the baseline simulation, and for the 4 sensitivity simulations with different BNF, fertilizer inputs, and LULUC. Lastly, we created additional 1000 different total land N pollution estimates by excluding river organic N exports. These provided a total of 6000 permutations.

A N_2O fraction of denitrification emissions can vary significantly in different climate, land use, and time (See Supplementary Materials in Bai and colleagues[33]). Global budgets[4,19-20,22], however, suggest large scale characteristic values ~0.08-0.11. For a N_2O fraction of soil and freshwater denitrification, we assigned different intervals: (0, 0.2) for global land and (0, 0.3) for tropical land. The higher upper bound for tropical land was because natural tropical systems have been recognized as a major hotspot of N_2O emissions[33-34].

A primary source of N_2 emissions associated with our net harvest are emissions from manure storage systems and wastewater treatment plants. Bouwman and colleagues[19] estimated denitrification (N_2O+N_2) emissions from manure storage systems and wastewater treatment plants as 8 TgN yr^{-1}, which accounts for a 0.09 fraction of our net harvest. For fractions of the net harvest into N_2 emissions, we assigned an interval (0, 0.2) for both global and tropical lands. We note that N_2O emissions from these sectors were assumed to be minor relative to N_2 emissions, based on the literature[35] that estimated N_2O emissions from wastewater treatment plants as 0.3 TgN yr^{-1}.

Zaehle[22] estimated human appropriation of N as 15 TgN yr^{-1}, which accounts for a 0.17 fraction of our net harvest. For fractions of human appropriation of the net harvest into durable goods, we assigned an interval (0.1, 0.3) for both global and tropical lands.

Supplementary Note 3. Comparison of simulated and published C balances in various tropical systems. Tropical NLI, estimated as approximately 1 (0.97-0.99) during 1992-1996 (Figure 3a), suggests that tropical land as a whole (including all kind of land use and land cover, such as agricultural lands, intact and disturbed forests) is nearly N neutral. This result aligns with filtered inverse models against an additional observational constraint, suggesting nearly neutral net C fluxes from tropical land for the same period (1992-1996)[36]. A recent study of plot measurements in Amazonian intact forests demonstrated a long-term increase in the aboveground biomass density since 1983 (~0-3 Mg ha^{-1} yr^{-1})[8], and a similar pattern during 1983-2005 was captured in our simulation (Supplementary Figure 11). For the same period (1983-2005), however, our simulation suggests that tropical forests as a whole (including both intact and disturbed forests) are a net C source of 271 TgC yr^{-1}, based on changes in aboveground C storage. This result appears to be consistent with a recent satellite-data-based study[37], demonstrating a net C source of 425 TgC yr^{-1} from tropical forests during 2003-2014. However, we note that this comparison cannot be done more formally, because of unconsidered terms in Baccini and colleagues[37]' approach, such as herbaceous and nonwoody vegetation, and because our simulations do not span the entire Baccini and colleagues[37]' period. Analyses of our simulations were limited up to 2005, because the used CMIP5 dataset for land-use changes stops in 2005[7].

Supplementary Note 4. Lake N cycle. Lakes receive dissolved organic N, ammonium, and nitrate plus nitrite from river inflows, and lose those by outflows to rivers and denitrification. Microbial processes in lakes are simulated by first-order loss function with respect to lake N content and with an adjustment for the influence of lake water temperature. Reported nonlinear regression function based on Lotic Intersite Nitrogen experiment reach-scale measurements[28,38-39] was adopted to estimate reaction rate constants of lake denitrification. The lowest measured value (0.034 day^{-1}) was set as a minimum reaction rate constant of lake denitrification (Supplementary Table 3). A maximum reaction rate constant of lake denitrification (0.05 day^{-1}) was calibrated to match reported and simulated river N exports. Previously used reaction rate constants of river mineralization and nitrification and river temperature reduction function[24] were also used for those of lakes.

$$\frac{dL_{DON}}{dt} = F_{DON}^{in} - F_{DON}^{out} - f_T'' k_{min}'' L_{DON} \qquad \text{Supplementary Eq. (1)}$$

$$\frac{dL_{NH_4^+}}{dt} = F_{NH_4^+}^{in} - F_{NH_4^+}^{out} + f_T'' k_{min}'' L_{DON} - f_T'' k_{nit}'' L_{NH_4^+} \qquad \text{Supplementary Eq. (2)}$$

$$\frac{dL_{NO_3^-}}{dt} = F_{NO_3^-}^{in} - F_{NO_3^-}^{out} + f_T'' k_{nit}'' L_{NH_4^+} - f_T'' k_{denit}'' L_{NO_3^-} \qquad \text{Supplementary Eq. (3)}$$

$$f_T'' = T_p''^{(T''-T_r'')} \qquad \text{Supplementary Eq. (4)}$$

$$k_{denit}'' = min\left\{k_{denit,max}'', max\left\{k_{denit,min}'', C_{d,s}\left(b_0 C_{NO_3^-}^{b_1} H^{b_2} c^t\right)\right\}\right\} \qquad \text{Supplementary Eq. (5)}$$

where i is DON, NH_4^+, and NO_3^-; L_i is lake N content (kg m^{-2}); F_i^{in} and F_i^{out} are river inflows to lakes and river outflows from lakes (kg m^{-2} s^{-1}); f_T'' is lake temperature reduction function; T_p'' is a parameter; T_r'' is a reference lake temperature (°C); T'' is lake water temperature (°C); k_{min}'', k_{nit}'', and k_{denit}'' are lake mineralization, nitrification, and denitrification (s^{-1}); $k_{denit,min}''$ and $k_{denit,max}''$ are minimum and maximum reaction rate constants of lake denitrification (s^{-1}); $C_{NO_3^-}$ is lake nitrate-N concentration (μmol L^{-1}); H is lake depth (m); b_0, b_1, and b_2 are constants; c^t is a log re-transform bias correction factor; $C_{d,s}$ is a unit-conversion constant (day s^{-1}).

Supplementary References

1. Harrison, J. A., Caraco, N. & Seitzinger, S. P. Global patterns and sources of dissolved organic matter export to the coastal zone: Results from a spatially explicit, global model. *Global Biogeochem. Cy.* **19,** GB4S04 (2005).
2. Meybeck, M., & Ragu, A. *River Discharges to the Oceans: An Assessment of Suspended Solids, Major Ions, and Nutrients* Environment Information and Assessment Technical Report (U.N. Environ. Programme, Nairobi 1996).
3. Dumont, E., Harrison, J., Kroeze, A. C., Bakker, E. J. & Seitzinger, S. P. Global distribution and sources of dissolved inorganic nitrogen export to the coastal zone: Results from a spatially explicit, global model. *Global Biogeochem. Cy.* **19,** GB4S02 (2005).
4. Galloway, J. N. et al. Nitrogen cycle: past, present and future. *Biogeochemistry* **70,** 153–226 (2004).
5. Green, P. A. et al. Pre-industrial and contemporary fluxes of nitrogen through rivers: A global assessment based on typology. *Biogeochem.* **68,** 71–105 (2004).
6. Lu, C. & Tian, H. Global nitrogen and phosphorus fertilizer use for agriculture production in the past half century: shifted hot spots and nutrient imbalance. *Earth Syst. Sci. Data* **9,** 181–192 (2017).
7. Hurtt, G. C. et al. The underpinnings of land-use history: three centuries of global gridded land-use transitions, wood-harvest activity, and resulting secondary lands. *Glob. Change Biol.* **12,** 1208–1229 (2006).
8. Brienen, R. J. W. et al. Long-term decline of the Amazon carbon sink. *Nature* **519,** 344–348 (2015).
9. Lamarque, J. F. et al. Multi-model mean nitrogen and sulfur deposition from the Atmospheric Chemistry and Climate Model Intercomparison Project (ACCMIP): evaluation of historical and projected future changes. Atmos. *Chem. Phys.* **13,** 7997–8018 (2013).
10. U.S. Environmental Protection Agency (USEPA). *Chesapeake Bay Phase 5.3 Community Watershed Model* EPA 903S10002 - CBP/TRS-303-10 (U.S. Environmental Protection Agency, Annapolis, 2010).
11. Herridge, D. F., Peoples, M. B. & Boddey, R. M. Global inputs of biological nitrogen fixation in agricultural systems. *Plant Soil* **311,** 1–18 (2008).
12. Vitousek, P. M., Menge, D. N., Reed, L. S. C. & Cleveland, C. C. Biological nitrogen fixation: Rates, patterns, and ecological controls in terrestrial ecosystems. *Philos. Trans. R. Soc. London B* **368,** 20130119 (2013).
13. Cleveland, C. C. et al. Global patterns of terrestrial biological nitrogen (N_2) fixation in natural ecosystems. *Global Biogeochem. Cy.* **13,** 623–645 (1999).
14. Cleveland C. C. et al. Patterns of new versus recycled primary production in the terrestrial biosphere. *Proc. Natl Acad. Sci. USA* **110,** 12733–12737 (2013).
15. Bouwman, L. et al. Exploring global changes in nitrogen and phosphorus cycles in agriculture induced by livestock production over the 1900–2050 period. *Proc. Natl Acad. Sci. USA* **110,** 20882–20887 (2013).
16. Galloway et al. Transformation of the nitrogen cycle: recent trends, questions, and potential solutions. *Science* **320,** 889–892 (2008).
17. Mayorga, E. et al. Global nutrient export from WaterSheds 2 (NEWS 2): Model development and implementation. *Environ. Model. Software* **25,** 837–853 (2010).
18. Seitzinger, S. P., et al., 2010: Global river nutrient export: A scenario analysis of past and future trends. *Global Biogeochem. Cy.* **24,** GB0A08.
19. Bouwman, A. F., et al. Global trends and uncertainties in terrestrial denitrification and N_2O emissions. *Philos. Trans. R. Soc. London Ser. B* **368,** 20130112 (2013).
20. Ciais, P. C. et al. Carbon and other biogeochemical cycles. In *Climate Change 2013: The Physical Science Basis* (eds. Stocker, T.F. et al.) 465–570 (Cambridge Univ. Press, Cambridge, 2013).
21. Dentener, F. et al. The global atmospheric environment for the next generation. *Environ. Sci. Technol.* **40,** 3586–3594 (2006).

22. Zaehle, S. Terrestrial nitrogen–carbon cycle interactions at the global scale. *Phil. Trans. R. Soc. B.* **368,** 20130125 (2013).
23. Post, W. M., Pastor, J., Zinke, P. J. & Stangenberger, A. G. Global patterns of soil nitrogen storage. *Nature* **317,** 613–616 (1985).
24. Lee, M., Malyshev, S., Shevliakova, E., Milly, P. C. D. & Jaffé, P. R. Capturing interactions between nitrogen and hydrological cycles under historical climate and land use: Susquehanna Watershed analysis with the GFDL Land Model LM3-TAN. *Biogeosciences* **11,** 5809–5826 (2014).
25. Gerber, S., Hedin, L. O., Oppenheimer, M., Pacala, S. W. & Shevliakova, E. Nitrogen cycling and feedbacks in a global dynamic land model. *Global Biogeochem. Cy.* **24,** GB1001, doi:10.1029/2008GB003336 (2010).
26. Parton, W. J. et al. Observations and modeling of biomass and soil organic matter dynamics for the grassland biome worldwide. *Global Biogeochem. Cy.* **7,** 785–809 (1993).
27. Cernusak, L. A., Winter, K. & Turner, B. L. Leaf nitrogen to phosphorus ratios of tropical trees: experimental assessment of physiological and environmental controls. *New Phytol.* **185,** 770–779 (2010).
28. Alexander, R. B. et al. Dynamic modeling of nitrogen losses in river networks unravels the coupled effects of hydrological and biogeochemical processes. *Biogeochem.* **93,** 91–116 (2009).
29. Wade, A. J. et al. Nitrogen model for European catchments: INCA, new model structure and equations. *Hydrol. Earth Syst. Sci.* **6,** 559–582 (2002).
30. Van Drecht, G., Bouwman, A. F., Harrison, J. & Knoop, J. M. Global nitrogen and phosphate in urban wastewater for the period 1970 to 2050. *Global Biogeochem. Cy.* **23,** GB0A03, doi:10.1029/2009GB003458 (2009).
31. Gruber, N. & Galloway, J. N. An Earth-system perspective of the global nitrogen cycle. *Nature* **451,** 293–296 (2008).
32. Sipler, R. E. & Bronk, D. A. *Biogeochemistry of Marine Dissolved Organic Matter Ch. 4* (Academic Press, 2014).
33. Bai, E. B., Houlton, Z. & Wang, Y. P. Isotopic identification of nitrogen hotspots across natural terrestrial ecosystems. *Biogeosciences* **9,** 3287–3304 (2012).
34. Bouwman, A. F., Fuag, I., Matthews, E. & John, J. Global analysis of the potential for N_2O production in natural soils. *Global Biogeochem. Cy.* **7,** 557–597 (1993).
35. Tian H. et al. The terrestrial biosphere as a net source of greenhouse gases to the atmosphere. *Nature* **531,** 225–228 (2016).
36. Stephens, B. B. Weak northern and strong tropical land carbon uptake from vertical profiles of atmospheric CO_2. *Science* **316,** 1732–1735 (2007).
37. Baccini, A. et al. Tropical forests are a net carbon source based on aboveground measurements of gain and loss. *Science* **358,** 230–234 (2017).
38. Mulholland, P. J. et al. Stream denitrification across biomes and its response to anthropogenic nitrate loading. *Nature* **452,** 202–205 (2008).
39. Mulholland, P. J. et al. Nitrate removal in stream ecosystems measured by ^{15}N addition experiments: denitrification. *Limnol. Oceanogr.* **54,** 666–680 (2009).
